# Direct quantification of topological protection in symmetry-protected photonic edge states at telecom wavelengths

**DOI:** 10.1038/s41377-020-00458-6

**Published:** 2021-01-06

**Authors:** Sonakshi Arora, Thomas Bauer, René Barczyk, Ewold Verhagen, L. Kuipers

**Affiliations:** 1 Kavli Institute of Nanoscience, Delft University of Technology, 2600 GA Delft, The Netherlands; 2 Center for Nanophotonics, AMOLF, Science Park 104, 1098 XG Amsterdam, The Netherlands

**Keywords:** Sub-wavelength optics, Nanophotonics and plasmonics, Interference microscopy, Scanning probe microscopy

## Abstract

Topological on-chip photonics based on tailored photonic crystals (PhCs) that emulate quantum valley-Hall effects has recently gained widespread interest owing to its promise of robust unidirectional transport of classical and quantum information. We present a direct quantitative evaluation of topological photonic edge eigenstates and their transport properties in the telecom wavelength range using phase-resolved near-field optical microscopy. Experimentally visualizing the detailed sub-wavelength structure of these modes propagating along the interface between two topologically non-trivial mirror-symmetric lattices allows us to map their dispersion relation and differentiate between the contributions of several higher-order Bloch harmonics. Selective probing of forward- and backward-propagating modes as defined by their phase velocities enables direct quantification of topological robustness. Studying near-field propagation in controlled defects allows us to extract upper limits of topological protection in on-chip photonic systems in comparison with conventional PhC waveguides. We find that protected edge states are two orders of magnitude more robust than modes of conventional PhC waveguides. This direct experimental quantification of topological robustness comprises a crucial step toward the application of topologically protected guiding in integrated photonics, allowing for unprecedented error-free photonic quantum networks.

## Introduction

The emergence of photonic topological insulators (PTIs) has led to promising theoretical and experimental approaches for topology-protected light–matter interactions^1^ and the integration of robust quantum devices^2^. Topologically protected photonic edge states offer robust energy transport with unprecedented guiding capabilities, providing a cornerstone for the efficient distribution of classical and quantum information in dense networks^3^. Usually, realizing nanophotonic systems with low backscattering at sharp bends is a great design challenge owing to the need to strike a balance between high bandwidth, low reflectance, and modest footprint. The promise of topologically protected photonic states supporting unhindered transport around defects and sharp corners without the need for optimization is thus especially interesting for on-chip applications. In addition to Chern-type PTIs that break time-reversal symmetry^3–6^, a time-reversal invariant realization of lossless optical transport was introduced theoretically on a dielectric photonic crystal (PhC) platform at telecom frequencies^7,8^. Although the existence of these states has been evidenced in the linear^9,10^ and nonlinear regimes^11^ and topological robustness has been inferred by high transmission^12,13^, quantifying their defining quality of scattering-free propagation has remained elusive. Potential interference effects and out-of-plane scattering losses at local disorder render this quantification challenging.

Here, we report a rigorous robustness evaluation of valley photonic edge eigenstates at telecom wavelengths. Local investigation of the states’ transport properties via phase-resolving near-field microscopy provides direct insight into topological protection through the distinction between forward and backward waves. We find that the examined edge states are two orders of magnitude more robust than modes in conventional waveguides. This determination of significantly suppressed back-reflection provides an essential step towards implementing topological guiding in on-chip photonic networks.

We realize valley-Hall PhCs (VPCs), which rely on the valley degree of freedom linked to the breaking of a specific lattice symmetry^12,14–16^. Similar to the valley-selective polarization caused by spin-orbit coupling in transition metal dichalcogenides^17^, these PhC lattices exhibit a non-vanishing Berry curvature at the *K* and K′ points of the Brillouin zone^18^. In contrast to the quantum spin-Hall effect emulating PhCs that support edge states at the Γ-point, the edge states in the following VPCs occur below the light line and thus feature negligible radiative losses. As each valley is associated with an intrinsic magnetic moment, the valley-Chern invariant $C_{K,K^{\prime}} = \pm \frac{1}{2}$ signifies a *pseudospin*^19^, rendering the bulk band structure topologically non-trivial. A domain wall formed by two parity-inverted copies of the PhC lattice results in two degenerate and robust edge-state eigenmodes confined to the interface that linearly traverse the photonic band gap (PBG), each with a unique *pseudospin*^20^. As long as the lattice symmetry is preserved and no inter-valley scattering occurs to flip the *pseudospin*, these edge states are predicted to be immune to reflection from local disorder along the domain wall^18,21^.

**Fig. 1:**
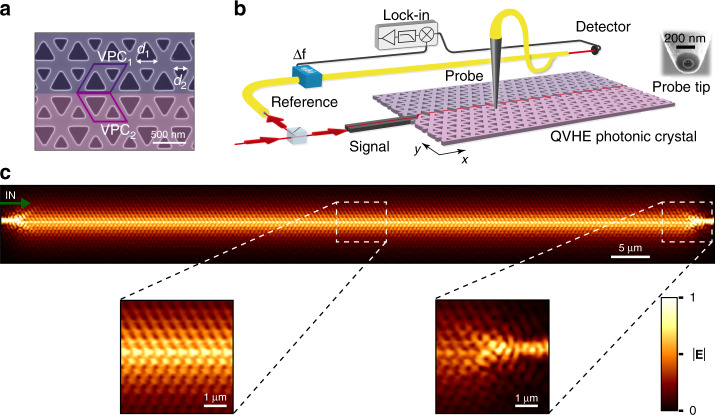
Experimental visualization of a topological edge state in a valley photonic crystal. **a** SEM of the fabricated structure with two pseudo-colored regions depicting the two lattices VPC_1_ and VPC_2_ with opposite valley-Chern invariants. The unit cell with lattice constant *a* = 503 nm consists of equilateral triangular holes of side lengths *d*_1_ = 0.7*a* and *d*_2_ = 0.45*a*. Scale bar: 500 nm. **b** Schematic of the near-field scanning optical microscope used to map the in-plane field distribution of the topologically non-trivial PhC edge mode. To facilitate heterodyne-based phase detection, the input beam is split into two branches, labeled signal and reference. An aperture-based near-field probe collects part of the evanescent tail of the in-plane field components while scanning over the crystal at a height of 20 nm and couples the collected light to a single-mode optical fiber. Inset: SEM image of the probe. **c** Measured normalized amplitude of the in-plane field components at a laser excitation wavelength of *λ* = 1600 nm over the extent of 165 unit cells, with the scale bar corresponding to 5 μm. Light is launched from a feed waveguide at the left side of the crystal, with the direction indicated by the red arrow. Left inset: zoomed-in view of the detected field amplitude pattern along the domain wall. Right inset: zoomed-in view of the out-coupling flank of the access waveguide

To determine the experimentally achievable robustness against backscattering, we fabricate a VPC working at telecom wavelengths on a silicon-on-insulator (SOI) platform following the design of ref. ^12^ (see Fig. 1). Light is coupled into the PhC structure in the +*x* direction from an access waveguide. This system supports edge modes of opposite group velocity ±*v*_*g*_ (see Supplementary Fig. S1) propagating along the domain wall between two parity-transformed lattices (VPC_1_ and VPC_2_). We visualize the spatial wavefunction of the mode with a phase-sensitive near-field scanning optical microscope (NSOM) (Fig. 1b)^22,23^. Figure 1c shows the measured two-dimensional in-plane field amplitude map at a wavelength of *λ* = 1600 nm. The detected transverse-electric (TE)-like field pattern confined to the interface of VPC_1_ and VPC_2_ extends laterally over roughly five unit cells, revealing an intricate sub-wavelength mode structure (left inset of Fig. 1c). The measured fields show close correspondence to the numerical calculations (see Supplementary Fig. S2). At the locations of the access and exit waveguides, the influence of broken lattice symmetry and the adjacent feed waveguide becomes evident in the distorted field pattern (right inset of Fig. 1c).

**Fig. 2:**
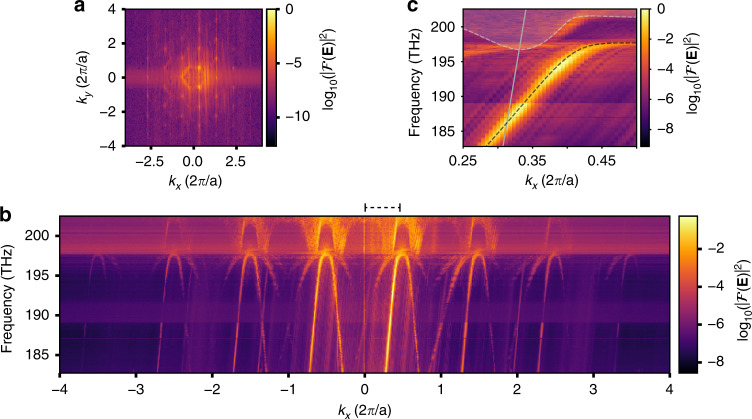
Momentum space of the VPC edge state. **a** Two-dimensional Fourier transform of the real-space amplitude distribution of the PhC mode. High-intensity points are periodically separated by the reciprocal lattice vector 2*π*/*a* in the direction of propagation *k*_*x*_ along the edge and by 4*π*/√3*a* in the transverse direction *k*_*y*_, representing the bulk reciprocal symmetry. **b** Experimentally retrieved dispersion diagram. Bright lines of positive slope indicate positive group velocity (forward-propagating modes), while lines with negative slope indicate a negative group velocity (backward-propagating modes). Consecutive Bloch harmonics are separated by the size of a single Brillouin zone (2*π*/*a*). Frequencies above 197.5 THz correspond to bulk bands. The Fourier intensity for each excitation frequency is normalized to the overall maximum value. In addition to the dominant modes in the forward and backward directions, lines with half and a third of the dispersion slope appear. These are attributed to a nonlinear interaction with the scanning near-field probe (see Supplementary Sec. III). **c** A close-up of the experimentally retrieved dispersion diagram limited to the first Brillouin zone is denoted by black dashed brackets in **b**. The black dashed lines indicate the numerically simulated values for the edge state (see Methods for details). The solid gray line denotes the light line and the gray dashed lines with grayed-out regions indicate the onset of the bulk bands

The heterodyne detection configuration of the employed NSOM gives access to the complex in-plane optical fields of the edge mode^24^. As a direct consequence of Bloch’s theorem, the two-dimensional spatial Fourier transformation ${\cal{F}}(k_x,k_y)$ of the measured field amplitude allows the individual analysis of Fourier components with positive and negative phase velocities. An illustrative Fourier map at *λ* = 1600 nm is displayed in Fig. 2a. By repeating the near-field scans and corresponding Fourier analysis for *λ* = [1480 nm–1640 nm] and integrating ${\cal{F}}(k_x,k_y)$ over *k*_*y*_, we extract the mode dispersion shown in Fig. 2b. We resolve at least six parallel lines due to the excellent signal-to-background ratio (S/B) of ~56 dB. The numerically simulated edge and bulk bands show excellent overlap with the experimentally measured dispersion, as seen in the overlaid enlarged view presented in Fig. 2c. The achieved spatial resolution, combined with the high S/B, enables us to resolve higher-order Bloch harmonics over multiple Brillouin zones. The lines with a positive slope correspond to a single forward-propagating mode with group velocity *v*_*g*_ = c/6. Closer inspection reveals negatively sloped lines corresponding to a single backward-propagating mode with group velocity −*v*_*g*_^25,26^. This separation of forward- and backward-propagating Bloch modes allows the local monitoring of backscattering along the domain wall.

Using this local phase and amplitude information, we probe a straight edge domain wall, as shown in Fig. 1c. We obtain the quantities *W*_*F*_ and *W*_*B*_ representing the forward and backward energy, respectively, through integration of their corresponding Fourier intensities. The ratio $\eta _e = \frac{{W_B}}{{W_F}} \approx 0.03$ unambiguously yields the conversion from forward to backward propagation, a result of scattering events occurring at and beyond the VPC end facet. Thus, *η*_*e*_ includes coupling of the forward to backward mode energy away from the topologically protected regime. This initial examination of the straight edge, with the observed back-propagation energy dominated by contributions of the end facet, calls for a more intricate analysis of topological protection.

**Fig. 3:**
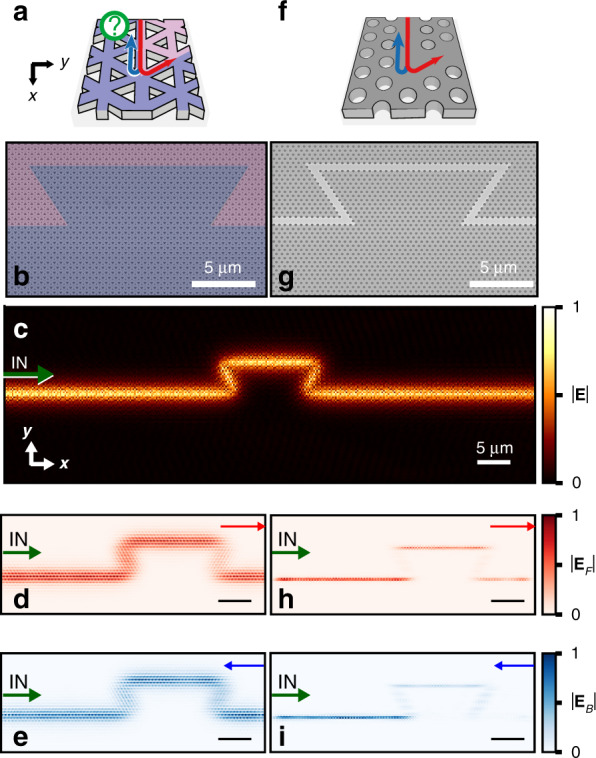
Directional transport along defects. For the topologically non-trivial VPC waveguide, **a** shows the schematic of the probed 120° corner. **b** Top-view SEM image of the fabricated Ω-shaped defect. Two-dimensional real-space amplitude maps showing the **c**, full mode amplitude distribution, **d** forward-propagating mode amplitude only, and **e** backward-propagating mode amplitude only. The amplitude maps are normalized independently to their maximum value. For a topologically trivial W1 waveguide, **f** schematically shows the mode propagation around a 120° corner, and **g** shows a top-view SEM image of the device. The two-dimensional amplitude maps of the filtered forward- and backward-propagating modes are shown in **h** and **i**, respectively. The direction of in-coupling is indicated by the green arrows. All scale bars correspond to 5 μm

To quantify protection without the aforementioned contributions, we introduce a trapezoidal (Ω-shaped) structure along the domain wall comprising four sharp corners (Fig. 3). This structure is expected to be topologically protected as *n* × 120° bends respect the underlying *C*_3_ lattice symmetry. Reflections characterized by energy coupled between the degenerate forward (*F*) and backward (*B*) propagating modes are indicated by red and blue arrows, respectively, in schematic Fig. 3a, with the fabricated structure displayed in Fig. 3b. Figure 3c shows a map of the measured amplitude of the VPC edge mode. By first separating the forward and backward modes through Fourier filtering in k-space based on the phase velocity of the edge mode and then performing an inverse Fourier transform, we obtain Fig. 3d, e. Figure 3d qualitatively demonstrates that the forward-propagating mode exhibits a near-unity transmission through the bend. The constant amplitude of the backward-propagating mode (Fig. 3e) also indicates near-unity transmission. This establishes that we may attribute the coupling of the forward and backward modes to the termination of the exit PhC waveguide. Put differently, light is perfectly guided around the Ω-shaped domain wall, with the transmission being independent of the presence of the defect itself.

**Fig. 4:**
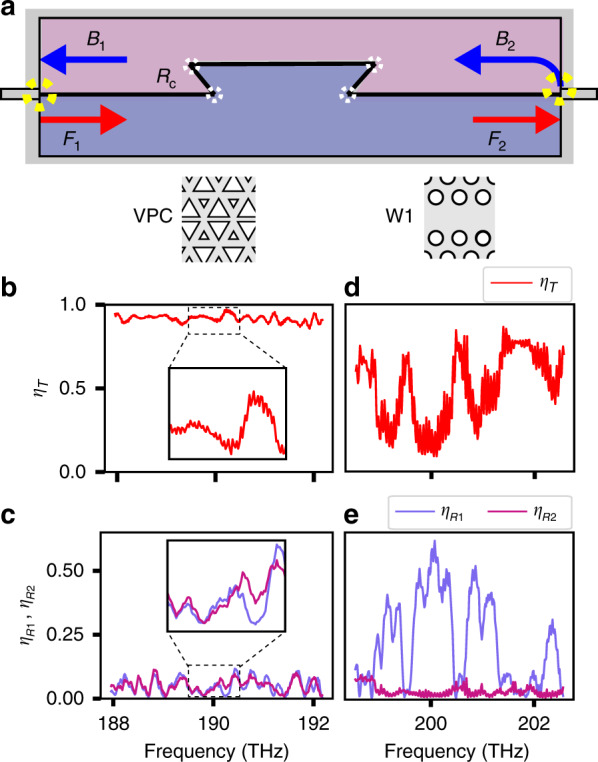
Degree of topological protection. **a** Schematic of the mode contributions in a Ω-shaped defect VPC waveguide. Red arrows indicate the forward-propagating modes, with *F*1 and *F*2 denoting the modes before and after the defect, respectively. Blue arrows indicate backward-propagating modes before (*B*1) and after (*B*2) the defect. Yellow dashed circles show the locations of in- and out-coupling facets. White dashed circles indicate the four 120° corners. **b** Plot of the transmission coefficient *η*_*T*_, with the inset demonstrating transmission over a small region ([189.5 THz–190.5 THz]). **c** Backward/forward energy ratio before (*η*_*R*1_) and after (*η*_*R*2_) the Ω-shaped defect in the VPC domain wall. The inset shows how the back-propagation energies before and after the defect are almost indistinguishable over the considered frequency range. **d** and **e** show the corresponding plots of **b** and **c** for the W1 waveguide, respectively

This observation is quantified by translating the locally measured amplitudes into mode energy ratios. We filter the Fourier intensity distribution to obtain the forward and backward-propagating mode energy before (*W*_*F*1_,*W*_*B*1_) and after (*W*_*F*2_,*W*_*B*2_) the Ω-bend (see Fig. 4a). Locally determined transmission through the defect for the linear part of the dispersion is shown in Fig. 4b. A mean transmission value *η*_*T*_ = *W*_*F*1_/*W*_*F*2_ of 0.92 is obtained for the chosen frequency range. In addition, the mode energy ratios calculated for the regions before (*η*_*R*1_ = *W*_*B*1_/*W*_*F*1_) and after (*η*_*R*2_ = *W*_*B*2_/*W*_*F*2_) the defect are shown for a frequency range of 4THz in Fig. 4c. We notice that *η*_*R*1_(*f*) and *η*_*R*2_(*f*) are almost indistinguishable. This strongly suggests that the contribution of the four symmetry-protected corners to the back-propagation energy is insignificant with respect to backscattering at the end facet.

Although expected, one can appreciate that the remarkably large transmission over the mode’s full frequency range^13,18,27^ is reasonably atypical in comparison with a topologically trivial standard *W1* PhC waveguide (see Methods for fabrication details). We again introduce a trapezoidal structure in this PhC waveguide (Fig. 3f, g). It is worth mentioning that the fabricated *W1* waveguide corners are not optimized for unity transmission at any given frequency^28^. In stark contrast to the forward and backward modes for a VPC (Fig. 3d, e), the *W1* modes (Fig. 3h, i) show significant loss across the defect. Moreover, the normalized backward amplitude map in Fig. 3i demonstrates that the dominant reflections already occurred at the first 120° corner. The mode energy here is converted to a back-reflected wave and additionally experiences out-of-plane scattering loss. The *η*_*T*_ measured through the Ω-structure in the *W1* PhC, shown in Fig. 4d, is on average one-third the *η*_*T*_ observed for the VPC. The strong reflection from the first corner is confirmed by the *η*_*R*_ shown in Fig. 4e, where *η*_*R1*_ is four times higher than *η*_*R2*_ for certain frequencies in the *W1* PhC waveguide.

In addition to the back-reflection from the individual corners, the direct evaluation of the Ω-shaped defect is affected by other aspects: out-of-plane scattering losses, scattering at the end facet and interference owing to multiple reflections along the domain wall. We notice rapid oscillations in *η*_*R*1,2_(*f*) before and after the defect (Fig. 4c, e). To disentangle the backscattering contribution from the aforementioned effects, we consider the complex scalar mode amplitude of the Bloch wave at different points along the domain wall. With the assumption of a perfectly mirror-symmetric device, we treat the defect as a single effective interface in a transfer-matrix model (TMM). Using *η*_*R*_ and *η*_*T*_ as input parameters to the model, we quantify the mean reflectance $\bar R_c$ of the full defect. Details of the model and the precise extraction method can be found in Supplementary Sec. II. Applying the model to the data for the topologically protected edge states shown in Fig. 4b, c yields a mean effective reflectance for the full defect $\bar R_c = 0.002 \pm 0.001$ and an out-of-plane scattering loss $\bar A_c = 0.080 \pm 0.002$ for the topologically protected edge states. Furthermore, we determine the average single-corner reflectance $R_c^{{\mathrm{single}}} = 0.0007$ from the TMM (see Supplementary Sec. II C).

The same approach applied to the data in Fig. 4d, e for the *W1* PhC waveguide reveals a reflectance $\bar R_c = 0.191 \pm 0.010$, two orders of magnitude larger than that observed for the VPC, and an out-of-plane scattering coefficient $\bar A_c = 0.304 \pm 0.017$. These values for the *W1* structure are in close agreement with literature^29–31^. A topologically protected PhC lattice thus reduces the experimentally achievable back-reflection from individual sharp corners by two orders of magnitude over the entire frequency range of the edge state. We confirm this finding and the applicability of the introduced TMM using finite-difference time-domain simulations for the same lattice designs with increasing numbers of corners (see Supplementary Sec. IV), further corroborating the obtained experimental limits to topological protection. The observation that the numerically extracted corner reflectance for the VPC is even lower than the experimentally determined reflectance suggests that we measure the effect of C_3_ symmetry-breaking disorder in the fabricated structure.

In summary, a direct experimental quantification of topological protection in VPC-based PTIs at telecom frequencies was achieved by accessing the full complex wavefunction of the edge state via phase-resolved near-field microscopy. This allows for determination of the back-reflection from topologically protected defects as well as for quantification of the experimentally unavoidable out-of-plane scattering losses. We unambiguously determined an experimental upper limit to the backscattering contribution from symmetry-protected defects in PhC-based topological edge states. This evaluation opens a direct pathway towards applied quantum topological photonic networks for secure and robust communications.

## Methods

### Simulations

Numerical simulations were performed using MIT Photonic Bands^32^ with the in-plane field distributions and retrieved dispersion relation shown in the supplementary materials. To match the calculated edge state to the measured dispersion relation, the refractive index of silicon was chosen as *n* = 3.36. To account for the corner roundness arising from fabrication, a fillet of 42 nm radius was added to the triangular holes of lattice constant *a* = 503 nm. The unit cell consisted of equilateral triangles, with a larger triangle side length *d*_1 _= 0.7*a* and smaller triangle side length *d*_2_ = 0.45*a*.

In addition, finite-difference time-domain calculations (FDTD Solutions by Lumerical) were used to verify the intrinsic transmittance spectra through 120° bends in a *W1* PhC waveguide.

### Device fabrication

The PhC slab was fabricated on a SOI platform with a 220-nm thick silicon layer on a 3 μm buried oxide layer. The fabrication was performed in two steps. First, a positive electron-beam resist of thickness 240 nm (AR-P 6200.09) was spin-coated between a monolayer of adhesion reagent HMDS and a conductive layer of E-Spacer 300Z. Then, the PhC design was patterned in the resist using e-beam lithography on a Raith Voyager with 50kV beam exposure. The e-beam resist was developed in pentyl acetate/O-xylene/MIBK:IPA(9:1)/isopropanol, and the SOI chip subsequently underwent reactive-ion etching in HBr and O_2_. In the second step, the photo-lithography resist AZ1518 was patterned using a Suss MABA6 Mask Aligner to define a selective wet-etching window on the PhC. After development with AZ400K:H_2_O, the buried oxide layer was removed in an aqueous 5:1 solution of hydrofluoric acid. The PhC was then subjected to critical point drying before being mounted in the near-field optical microscopy setup.

The PhCs were terminated on both sides such that a TE single-mode Si-ridge waveguide was extended as a PhC waveguide into the crystal to enable better index matching for efficient in-coupling^12^.

The PhC lattice featured a honeycomb configuration of two equilateral triangles in a unit cell of lattice constant *a* = 503 nm. One triangle was scaled up (*d*_1_ = 0.7*a*), and the other down (*d*_2_ = 0.45*a*), while preserving *C*_3_ lattice symmetry. A domain wall was created along *VPC*_1_ and *VPC*_2_ by applying a parity operation along the spatial *y*-coordinate. Two different VPC domain walls were fabricated to facilitate transmission and backscattering comparisons. The straight edge VPC had dimensions 195*a* × 55*a*, designed such that the PBG fell within the tunable laser wavelength range of 1480 nm–1640 nm. For the trapezoidal edge VPC domain wall, the two diagonals extended over 12 unit cells, whereas the horizontal extent of the defect between the second and third corners was 34 unit cells.

The standard *W1* waveguide was formed from a honeycomb lattice of circular holes, with a lattice constant *a* = 420 nm and hole radius *r* = 120 nm, where one row of circular holes was removed.

### Near-field optical microscopy setup

The utilized aperture-based near-field optical microscope consisted of a tapered optical fibre coated homogeneously with 140 nm aluminum. An aperture of ca. 170 nm was created at its apex via focused ion beam milling. Scanning the probe over the silicon membrane at a relative height of ca. 20 nm, controlled via shear force feedback, resulted in the pickup of the local in-plane field components. Their amplitudes and phases were determined using a heterodyne detection scheme with the coherent reference light beam shifted by Δ*f* = 40 kHz in frequency^23^.

## Supplementary information

Supplementary File

## Data Availability

All data obtained in the study are available from the corresponding author upon reasonable request.
