# Supplementary material for: Direct quantification of topological protection in symmetry-protected photonic edge states at telecom wavelengths
Source: Light Sci Appl. 2021 Jan 6;10:9. doi: 10.1038/s41377-020-00458-6 (PMC7788078; doi:10.1038/s41377-020-00458-6)
Supplement: Supplementary file 1 — Supplementary File [file 41377_2020_458_MOESM1_ESM.pdf]

Supplementary information: Direct quantification of topological protection in photonic crystals supporting symmetry-protected edge states at telecom wavelengths

Sonakshi Arora,[1] Thomas Bauer,[1] René Barczyk,[2] Ewold Verhagen,[2] and L. Kuipers[1,*]

[1] *Kavli Institute of Nanoscience, Delft University of Technology, 2600 GA, Delft, The Netherlands*
[2] *Center for Nanophotonics, AMOLF, Science Park 104, 1098 XG Amsterdam, The Netherlands*

GLOSSARY

VPC — valley photonic crystal
TMM — transfer matrix model

I. NUMERICAL CALCULATION OF EDGE STATE DISPERSION AND EIGENMODES

The edge state eigenfrequencies $f(k)$ as well as eigenmodes $\mathbf{E}_k(\mathbf{r})$ that are localized at the domain wall between two mirror-symmetric valley photonic crystals (VPCs) were calculated for an in-plane wavevector k with the freely available MIT Photonic Bands solver [1]. It determines the Bloch eigenmodes of the full three dimensional photonic crystal structure using a plane-wave basis set and periodic boundary conditions. We used a simulation supercell of dimensions $a \times 28a \times 10h$, where h is the thickness of the free-standing silicon slab and a is the lattice constant of the VPC patterned therein. This supercell was sufficiently large to avoid interactions between neighbouring supercells in y and z. The calculations used an in-plane grid size of $a/32$ and out-of-plane grid size of $a/16$, which ensured convergence of the eigenvalues to better than 0.1%. To account for surface passivation effects of the experimentally employed silicon membrane of height $h = 220\,\mathrm{nm}$ and to adapt the resulting eigenfrequencies to the experimentally determined dispersion relation, the effective refractive index of silicon was modelled to be 3.36.

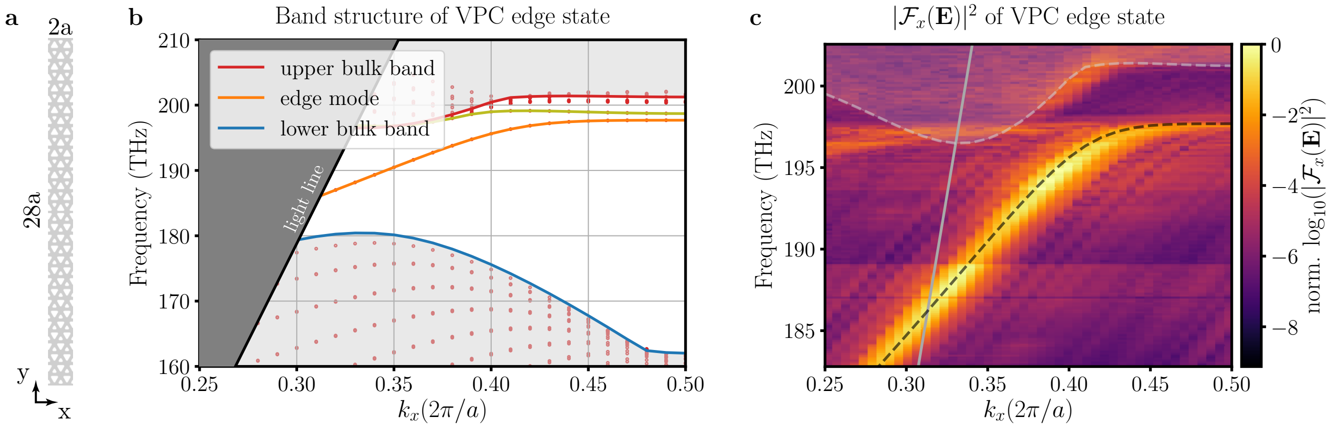

Figure S1. Band structure calculations. **a**, Geometry of the photonic crystal membrane used in the eigenmode calculations, with grey and white representing silicon and air, respectively. For better visibility, two unit cells are shown along the domain wall. **b** Numerically calculated band structure of the edge state eigenmode (orange) as well as the upper and lower bulk bands (red and blue, respectively) of the photonic crystal. The light cone is displayed as dark grey shading, while the bulk band region has been shaded light grey. In addition to the topologically non-trivial edge mode, there exists a second mode confined to the edge at large k_x, shown in ochre. **c** Comparison of the fundamental Brillouin zone of the experimentally retrieved dispersion relation and the numerically calculated edge state eigenfrequencies (black dashed line) as well as bulk bands (grey dashed line and shaded region), highlighting the excellent overlap between experiment and simulation. The light line is given by the solid grey line.

* Corresponding author: l.kuipers@tudelft.nl

The lattice constant was chosen as $a = 503\,\text{nm}$, and the base length of the triangular holes in each unit cell, determined from SEM images, was $d_1 = 0.70a$ and $d_2 = 0.45a$ for the large and small triangle, respectively. Both triangular holes were modeled with a corner rounding of $r = 42\,\text{nm}$ to account for fabrication-related deviations from their ideal structure. We construct a bridge-type domain wall between two mirror-symmetric sub-lattices VPC_1 and VPC_2 in the supercell, with the base of the large triangles facing each other. The periodic nature of the simulation subsequently dictates the additional occurrence of the complementary domain wall (base of small triangles facing each other) at the boundary of the supercell (see Fig. S1**a**). The calculated edge state eigenmodes located at this complementary domain wall were filtered out, and the resulting band structure for the topologically protected edge state as well as the bulk VPC eigenfrequencies determining the system's band gap are shown in Figure S1**b**. The latter were calculated for a single unit cell of one of the sub-lattices and subsequently mapped to the chosen interface orientation by projecting the resulting bulk frequencies onto the k_x direction. In addition to the edge state traversing the band gap with a linear dispersion around the K-point, a second mode confined to the central domain wall is found branching off the top bulk band for high in-plane k-values (shown in ochre in Fig. S1**b**). Comparing the calculated band structure to the fundamental Brillouin zone of the experimentally retrieved dispersion relation, an excellent agreement in both position of the band edge as well as edge state dispersion is found, with the mode dispersion of the edge state extending into the light cone for $k_x < 0.31 \cdot 2\pi/a$ (see Fig. S1**c**).

To verify the experimentally determined near-field structure of the edge mode (shown for a wavelength of $\lambda = 1600\,\text{nm}$ in Fig. S2**a**), the field distribution of the eigenstate was extracted from the simulation 20 nm above the silicon membrane. The in-plane field amplitude distribution is displayed for an illustrative k-point of $k_x = 0.32 \cdot 2\pi/a$ in Fig. S2**b**. The corresponding eigenfrequency of the edge state at this k-point is $f = 187.0\,\text{THz}$. It can be seen that the mode symmetries of this numerically determined eigenmode match for the mode's in-plane electric field amplitude and the experimentally measured mode distribution.

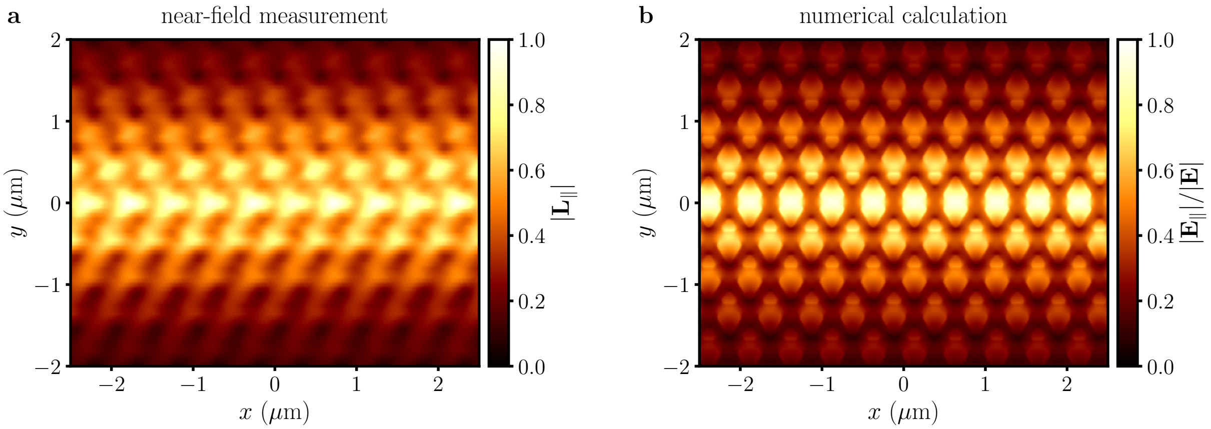

Figure S2. Comparison between **a**, the experimentally retrieved near-field amplitude map for $\lambda = 1600\,\text{nm}$ ($f = 187.4\,\text{THz}$) and **b**, the numerically calculated in-plane electric field distribution of the edge mode at $k_x/(2\pi/a) = 0.32$ ($f = 187.0\,\text{THz}$).

II. QUANTIFICATION OF CORNER REFLECTIVITY AND LOSS

Our main goal in this section is to quantify the back-scattering of topologically protected edge states – bound to a VPC domain wall – from an Ω-shaped defect of four 120° corners (cf. Fig. 3**b** of the main manuscript) by comparing the measured forward and backward propagating energies before and after the defect to an analytical model. We introduce a transfer matrix model (TMM) approach and compare its results to the back-scattering observed in a similarly shaped W1-type photonic crystal waveguide, which is based on one missing row of holes in a hexagonal lattice. We extract the reflection coefficient R_c^{single} of an individual corner in the waveguide as a quantitative measure of back-scattering, and quantify the associated loss A_c^{single} which is dominated by out-of-plane scattering.

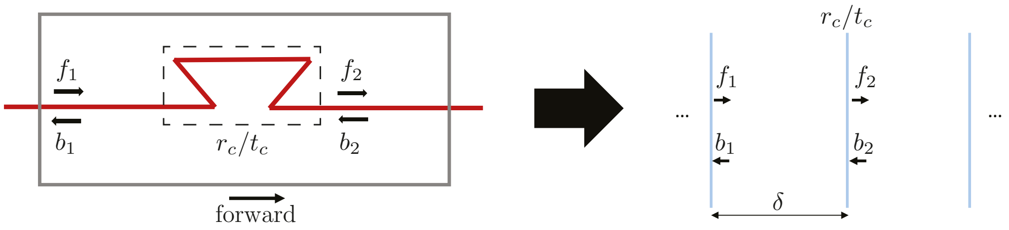

Figure S3. Sketch of the experimentally investigated VPC with an Ω-shaped domain wall defect, as well as its representation within a transfer matrix model. The effect of the Ω-shaped defect onto the field amplitudes is approximated by complex reflection and transmission coefficients r_c and t_c, and the distance between the detection points is associated with a propagation phase δ.

A. Transfer matrix model

We start from Bloch's theorem for a photonic crystal domain wall (or waveguide) with periodicity a along the x-direction, resulting in the Bloch state

$$\mathbf{E}_{k_x}(\mathbf{r}) = e^{\imath k_x x} \cdot \mathbf{u}_{k_x}(\mathbf{r}) \,, \quad \text{with } \mathbf{u}_{k_x}(\mathbf{r} + a\hat{\mathbf{e}}_x) = \mathbf{u}_{k_x}(\mathbf{r}), \tag{S1}$$

where k_x is the Bloch wavevector along the mode's propagation direction, and $\mathbf{u}_{k_x}(\mathbf{r})$ represents its full spatial field distribution.

To compare the mode at different positions along the domain wall, we separate a complex scaling amplitude a_k from the mode's periodic distribution, resulting in

$$\mathbf{E}_{k_x}(\mathbf{r}) = a_k e^{\imath k_x x} \cdot \mathbf{u'}_{k_x}(\mathbf{r}), \tag{S2}$$

with $\mathbf{u'}_{k_x}$ a fixed function, independent of the unit cell or time at which the mode field is evaluated. The full spatial information in each unit cell is thus effectively reduced to the scalar complex amplitude value a_k.

The complex amplitude together with the reciprocal nature of the employed photonic crystals enable us to describe back-scattering sites along the domain wall as interfaces with complex amplitude reflection and transmission coefficients r_c and t_c, respectively. Furthermore, we introduce a loss channel A_c, incorporating out-of-plane scattering losses, via

$$R_c + T_c = 1 - A_c \,, \tag{S3}$$

where $R_c = |r_c|^2$, $T_c = |t_c|^2$.

Without loss of generality, we consider the forward and backward propagating field amplitudes f_1, f_2 and b_1, b_2 to denote the value of a_k at specific positions on the sample as shown in Fig. S3, and get

$$b_1 = r_c f_1 \, e^{i2\delta} + t_c b_2 \, e^{i\delta} \tag{S4}$$

$$f_2 = t_c f_1 \, e^{i\delta} + r_c b_2 \,, \tag{S5}$$

with the propagation phase $\delta = k \cdot d$ given by the wavenumber k and distance d between the two detection points 1 and 2, which are placed immediately after an interface. Equations S4, S5 can be cast into the compact form

$$\begin{pmatrix} f_1 \\ b_1 \end{pmatrix} = \mathbf{M}_c \begin{pmatrix} f_2 \\ b_2 \end{pmatrix} \,, \tag{S6}$$

where

$$\mathbf{M}_c = \frac{1}{t_c} \begin{pmatrix} e^{-i\delta} & 0 \\ 0 & e^{i\delta} \end{pmatrix} \begin{pmatrix} 1 & -r_c \\ r_c & t_c^2 - r_c^2 \end{pmatrix} \tag{S7}$$

is the transfer matrix describing light propagation from position 1 to 2 in the multilayer stack.The propagation through a series of N layers can then be written as a single matrix $\tilde{M}$, given by

$$\tilde{\mathbf{M}} = \prod_{i=1}^{N} \mathbf{M}_i \,. \tag{S8}$$

Here, $\mathbf{M}_i$ are the transfer matrices of the individual layers (with corresponding distances and reflection/transmission coefficients).

It is straightforward to relate any field amplitudes in a TMM to each other by appropriate transfer matrix multiplications. The experimentally determined energy densities on which we base our analysis are given by $F_n = |f_n|^2$, $B_n = |b_n|^2$ $(n = 1, 2)$ and are repeated from Fig. 4**b-e** of the main manuscript in Fig. S4 over an excitation frequency window of ca. 4 THz. If we want to achieve a full overlap of these energy densities with predictions from the TMM model, we not only need to take into account the four 120° corners of the tailored topologically protected defect, but additionally all scattering sites after the defect. These include scattering at the transition from the VPC domain wall to an intermediate PhC waveguide, the subsequent transition to a silicon strip waveguide, and scattering at potential fabrication defects. Since these additional interfaces are not protected by the mirror symmetry of the VPC lattice, it is expected that their reflection coefficients dominate the entire system.

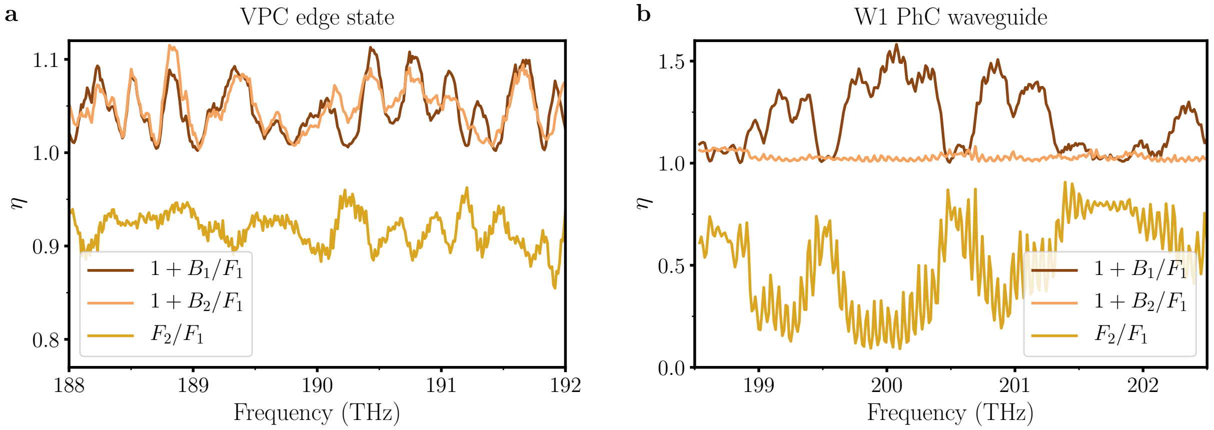

Figure S4. Experimentally retrieved energy of the forward (F_2) and backward (B_1, B_2) propagating modes before and after the Ω-shaped defect, normalized to F_1. The backward mode intensities are shifted by 1 for better visibility. **a**, VPC edge state. **b**, W1 waveguide.

B. Effective corner reflectivity

Our aim is to arrive at a relation between the measured energy densities before and after the defect, and the intensity reflection coefficient R_c and loss A_c. If we, in fair approximation, assume our devices to be mirror symmetric (as by design), reciprocity dictates equal coefficients for forward and backward propagation through the full Ω-shaped defect. We can thus combine the four individual corners into a single effective interface with complex amplitude reflection and transmission coefficients r_c and t_c (see Fig. S3). We divide (S4), (S5) by f_1 and take the absolute square to obtain

$$\begin{aligned}\frac{B_1}{F_1} &= R_c + \frac{B_2}{F_1}\,T_c + \left(\sqrt{R_c}e^{-i\theta_{r_c}}\,\sqrt{T_c}e^{i\theta_{t_c}}\,\sqrt{\frac{B_2}{F_1}}e^{i\theta_{b_2}}e^{-i\theta_{f_1}} + c.c.\right)\\ &= R_c + \frac{B_2}{F_1}\,T_c + 2\sqrt{R_c\,T_c\,B_2\,F_1}\,\cos(\theta_c - \theta) \qquad \text{(S9)}\\ \frac{F_2}{F_1} &= T_c + \frac{B_2}{F_1}\,R_c + 2\sqrt{R_c\,T_c\,\frac{B_2}{F_1}}\quad\cos(\theta_c + \theta)\,. \qquad \text{(S10)}\end{aligned}$$

Here, *c.c.* denotes the complex conjugate and we express complex quantities in their polar form ($z = \sqrt{Z}e^{i\theta_z}$), with the phase differences $\theta_c = \theta_{r_c} - \theta_{t_c}$ and $\theta = \theta_{b_2} - \theta_{f_1}$. Using (S3), we cast (S9), (S10) into the form

$$\frac{B_1 - B_2}{F_1 - B_2} = R_c - \frac{A_c B_2 - 2\sqrt{R_c(1 - R_c)F_1 B_2}}{F_1 - B_2}\,\cos(\theta_c - \theta) \qquad \text{(S11)}$$

$$\frac{F_1 - F_2}{F_1 - B_2} = R_c + \frac{A_c F_1 - 2\sqrt{R_c(1 - R_c)F_1 B_2}}{F_1 - B_2}\,\cos(\theta_c + \theta) \qquad \text{(S12)}$$

Equations (S11), (S12) represent the central result of the TMM analysis, and the corresponding data is presented in Fig. S5.

Due to the expected strong influence of scattering events from outside the topological photonic crystal, as seen by the strong correlation between the signals B_1/F_1 and B_2/F_1 in Fig. S4**a**, we utilize an effective averaging approach. Such an approach is based on the linear slope of the VPC edge state dispersion. We can then disentangle the reflection at the tailored Ω-shaped defect from any subsequent back-scattering events by averaging over a frequency range where the interference effects between the latter lead to cosine-like intensity modulations with multiple full periods. Both θ_c and θ in (S11), (S12) are oscillatory functions of frequency since they incorporate the propagation phases (cf. Eqs. S4, S5). Therefore, by applying a frequency average over the experimental range of ca. 4 THz (denoted by $\langle\cdot\rangle$) to (S11), (S12), we average out the cosine modulation and solve for the mean effective defect reflectivity and loss

$$\langle R_c \rangle = \frac{(\overline{B_1 - B_2})\overline{F_1} - (\overline{F_1 - F_2})\overline{B_2}}{\overline{F_1} + \overline{B_2}} \tag{S13}$$

$$\langle A_c \rangle = \frac{(\overline{F_1 - F_2}) - (\overline{B_1 - B_2})}{\overline{F_1} - \overline{B_2}} \,, \tag{S14}$$

where we defined the operator

$$\bar{\cdot} \equiv \left\langle \frac{\cdot}{F_1 - B_2} \right\rangle \,, \tag{S15}$$

and assume no correlation in the spectral behavior of A_c and F_1, B_2. The resulting averages are displayed as straight lines in Fig. S5, with their standard deviation indicated by the shaded regions.

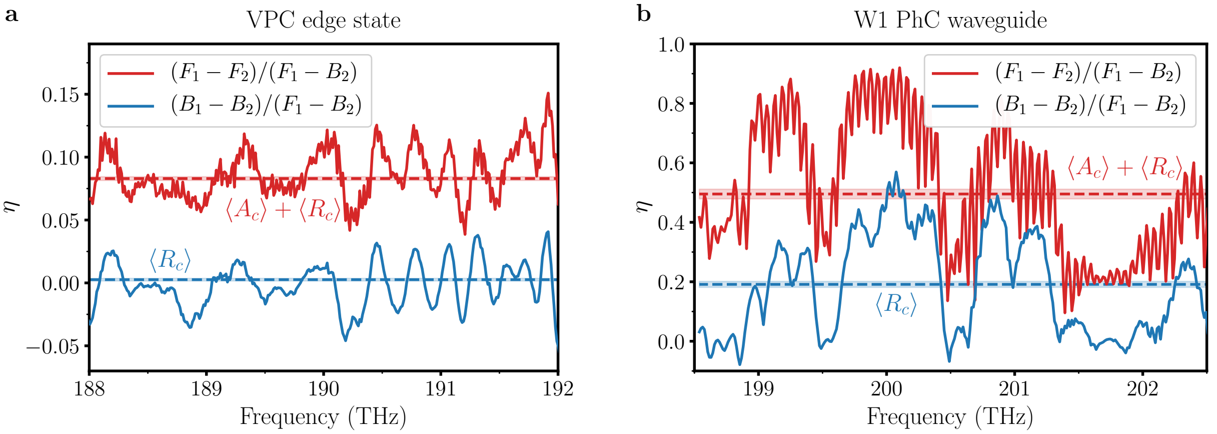

Figure S5. Evaluation of (S11), (S12) for the data shown in Fig. S4, where $\langle R_c \rangle$ and $\langle R_c \rangle + \langle A_c \rangle$ according to (S13), (S14) are indicated as red and blue dashed lines, respectively. The shaded region around the lines corresponds to the standard deviation of the extracted mean values. **a**, VPC edge state. **b**, W1 waveguide.

C. Single corner reflectivity

From the mean effective interface values $\langle R_c \rangle$ and $\langle A_c \rangle$ via (S13), (S14), we infer an estimate for the coefficients of the individual corners, i.e., when representing each of the four waveguide bends by a single interface in the TMM. The distances between the interfaces are set to the design parameters, as indicated in Fig. S3. We model a waveguide with four identical corners, then search for an $\langle R_c^{\mathrm{single}} \rangle$ such that the mean effective $\langle R_c \rangle$ given by (S13) reproduces the value extracted from the experiment. The single corner absorption is subsequently given by

$$\langle A_c^{\mathrm{single}} \rangle = 1 - \langle R_c^{\mathrm{single}} \rangle - \sqrt[4]{1 - \langle R_c \rangle - \langle A_c \rangle} \,. \tag{S16}$$

The mean effective parameters extracted from our measurements of the VPC domain wall and W1 waveguide are displayed in Fig. S5 and listed together with the single corner parameters in Tab. I. For the VPC edge state defect, we obtain $\langle R_c^{\mathrm{single}} \rangle \approx 0.1\%$ and $\langle A_c^{\mathrm{single}} \rangle \approx 2\%$. While the value of $\langle A_c^{\mathrm{single}} \rangle$ might seem large for a topological photonic

	VPC edge state	W1 waveguide
$\langle R_c \rangle$	0.0026 ± 0.0010	0.191 ± 0.010
$\langle A_c \rangle$	0.0804 ± 0.0014	0.304 ± 0.017
$\langle R_c^{\text{single}} \rangle$	0.0007	0.052
$\langle A_c^{\text{single}} \rangle$	0.0208	0.105

Table I. Comparison of extracted effective mean corner reflectivity and loss for the Ω-shaped defect within the studied photonic crystal waveguides, as well as the estimates for single corners.

insulator, topological protection is only given for in-plane propagation, with out-of-plane confinement in the silicon slab solely provided by total internal reflection. If we apply the described model to the experimentally measured mode amplitudes of a topologically trivial W1 photonic crystal waveguide, we obtain a corner reflectivity that is two orders of magnitude higher with respect to the topologically protected system ($\langle R_c^{\text{single}} \rangle \approx 5\%$), while we also see roughly a four-fold increase in out-of-plane scattering ($\langle A_c^{\text{single}} \rangle \approx 11\%$). Such an increase in losses matches well with the expected scaling behaviour with the modes' group velocity of v_g^{-2} [2], with $v_g \approx c/5.8$ for the VPC edge state and $v_g \approx c/10.1$ for the W1 waveguide mode.

In consequence, the extracted reduction of backreflection between the two waveguide systems highlights the inherent advantage of topological photonic systems even in experimentally achievable conditions.

III. DISPERSION RELATION OF W1 PHOTONIC CRYSTAL WAVEGUIDE

To compare the robustness against reflection from a corner defect between the VPC edge state and a W1-type photonic crystal waveguide, both modes need to be considered at comparable group velocities v_g, since loss scales with v_g^{-2} [2]. Thus, we experimentally determine the dispersion relation of the W1-type photonic crystal waveguide (analogous to the VPC edge state dispersion from the main manuscript, shown in Fig. 2**b**), and show the bands of forward and backward propagating Bloch modes spanning several Brillouin zones (BZs) in Fig. S6. One key observation here is that the forward propagating mode in the fundamental BZ is located at negative k_x, in accordance with the mode's expected behaviour [2]. In addition to the predicted band in forward and backward direction, bands with half and a third of the dispersion slope of the fundamental mode appear. These were already observed in a GaAs-based photonic crystal and attributed to a nonlinear interaction with the scanning near-field probe [3].

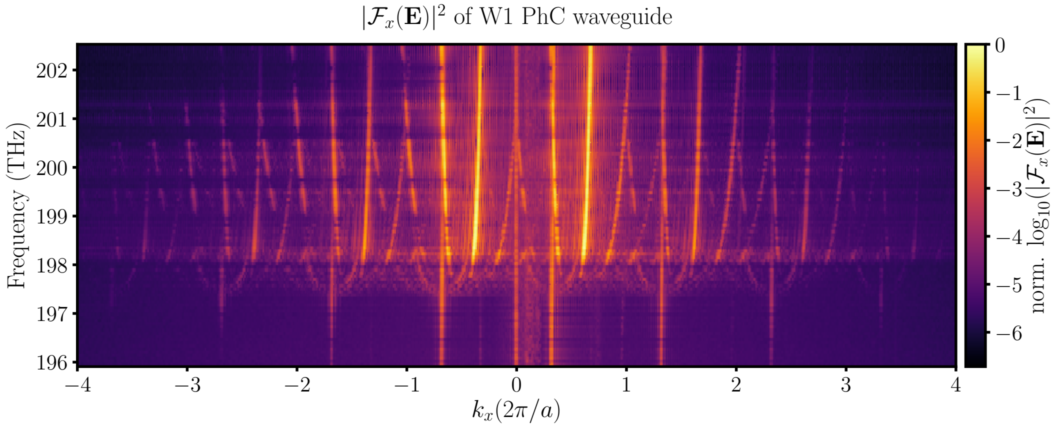

Figure S6. Dispersion relation of the fundamental mode of a W1-type photonic crystal waveguide, retrieved via Fourier transforms of near-field maps of the mode while scanning the excitation wavelength. The lattice constant of the investigated photonic crystal is $a = 420\,\text{nm}$, with a hole of radius $r = 120\,\text{nm}$ constituting the unit cell of the hexagonal lattice.

IV. INFLUENCE OF AMOUNT OF CORNERS ON BACKSCATTERING

In order to validate the experimental retrieval of the best estimate for a corner reflectivity from the introduced transfer matrix model, we calculated the back-reflected energy densities for different numbers of corners using finite difference time domain simulations (Lumerical FDTD). We construct a VPC interface and W1 photonic crystal

waveguide with the same dimensions and a similar in-coupling scheme as used in the experiment. The lattice constant a and sizes of the triangular holes in the unit cell of the VPC were thus chosen as $a = 500\,\text{nm}$, $d_1 = 0.70a$ and $d_2 = 0.45a$, while the W1 waveguide was constructed with a lattice constant of $a = 420\,\text{nm}$ and hole radius of $r = 120\,\text{nm}$. To reduce the computational cost for the simulations, 2D calculations were performed with effective refractive indices adapted to approximately match the experimentally retrieved band gaps around $190 - 200\,\text{THz}$. In this configuration, out-of-plane scattering is absent in the simulations in contrast to the experiment. We utilize a broadband source in the feed waveguide to launch a pulse with a spectral range of $1450\,\text{nm} - 1650\,\text{nm}$ and temporal width of $\Delta t \approx 40\,\text{fs}$. The simulation domain was encapsulated by perfectly matched layers (PMLs) with sufficient thickness to minimize unwanted reflections at the domain boundaries, and the simulation was performed for $10\,\text{ps}$ to allow for the pulse energy to adequately decay in the PMLs.

The in-plane electric field components over an area of 20×20 unit cells along the interface/waveguide before the corner(s) were subsequently extracted (see Fig. S7**a-c** for the different defect configurations) and used to calculate the mode energy of the forward (F_1) and backward (B_1) traveling edge state or waveguide mode via their dispersion relation, analog to the experimental realization. The resulting energy ratio $\eta_{R_1} = B_1/F_1$ is shown for the three different configurations in Fig.S7**a-c** on a logarithmic scale to compare the QVHE-emulating VPC and W1 PhC waveguide for each configuration, as well as in Fig. S7**d,e** on a linear scale to follow the evolution of the two modes with increasing amount of corners. In the case of two and four 120° corners, the field energies F_2, B_2 were additionally extracted from a similar area after the defect.

Looking at the variation in the energy ratio, we see that the oscillation amplitude of the back-reflected mode energy increases as the amount of interface corners is increased. In addition, the interference between the different corners leads to a distinct Fabry-Perot-type modulation pattern for the W1 waveguide (Fig. S7**e**), while an increase of contributing oscillation periods can be seen for the VPC edge (Fig. S7**d**). The nearly flat reflection coefficient for a single corner in the W1 waveguide of $\langle \eta_{R_c} \rangle = 0.288$ highlights the dominating influence of the W1 corner over other sources of backscattering, while the modulation seen in the VPC edge data for a single corner hints at a comparable backscattering strength from the corner and the PMLs terminating the simulation domain.

Evaluating the shown energy ratios via the transfer matrix model of section II, we get an effective full reflectivity of $\langle R_c \rangle = [0.2, 1.4, 3.9] \cdot 10^{-5}$ for a system containing 1, 2 and 4 corners along the VPC interface, respectively. The W1 waveguide system exhibits $\langle R_c \rangle = [0.288, 0.443, 0.571]$ for the same configurations. The relative low reflectivity for the VPC suggests that in addition to backscattering caused by the introduced defects, the backward propagating mode energy is affected by numerical as well as meshing artifacts in the simulations. These are negligible for the W1 simulations, which show a clear trend of increasing system reflectivity with number of corners. Using the reflectivity $\langle R_c \rangle$ for the W1 waveguide structures with 2 and 4 corners, we can estimate the single-corner reflectivity with the approach of section IIC. This results in $\langle R_c^{\text{single}} \rangle = [0.285, 0.284]$ for the two- and four-corner system. This is in good agreement with the reflectivity $\langle R_c \rangle = 0.288$ that was calculated directly for a single-corner system, corroborating the approach of section IIC to estimate single-corner from full-defect reflectivity as we perform in the main text.

The extracted four orders of magnitude difference in reflectivity between the VPC edge state and the W1 waveguide mode highlights the robustness of the former against the designed corner defects. While these 2D simulations achieve an additional two orders of magnitude decrease in backscattering as compared to the experiment, we want to emphasize here that no disorder in the position and size of the triangles constituting the VPC unit cells was taken into account in the simulations. Typical state-of-the-art manufacturing techniques allow for disorder as small as $\sigma \approx 1\,\text{nm}$ [4], which is an order of magnitude larger than the grid size chosen in the shown numerical calculations. The discrepancy between experimentally determined and numerically extracted corner reflectivity can thus be understood as an indirect measure of the C_3 symmetry-breaking disorder in the experimental system.

---

[1] S. Johnson and J. Joannopoulos, Block-iterative frequency-domain methods for Maxwell's equations in a planewave basis, Optics Express **8**, 173 (2001).

[2] R. J. P. Engelen, D. Mori, T. Baba, and L. Kuipers, Two Regimes of Slow-Light Losses Revealed by Adiabatic Reduction of Group Velocity, Physical Review Letters **101**, 103901 (2008).

[3] A. Singh, G. Ctistis, S. R. Huisman, J. P. Korterik, A. P. Mosk, J. L. Herek, and P. W. H. Pinkse, Observation of nonlinear bands in near-field scanning optical microscopy of a photonic-crystal waveguide, Journal of Applied Physics **117**, 033104 (2015).

[4] S. Mazoyer, P. Lalanne, J. Rodier, J. Hugonin, M. Spasenović, L. Kuipers, D. Beggs, and T. Krauss, Statistical fluctuations of transmission in slow light photonic-crystal waveguides, Optics Express **18**, 14654 (2010).

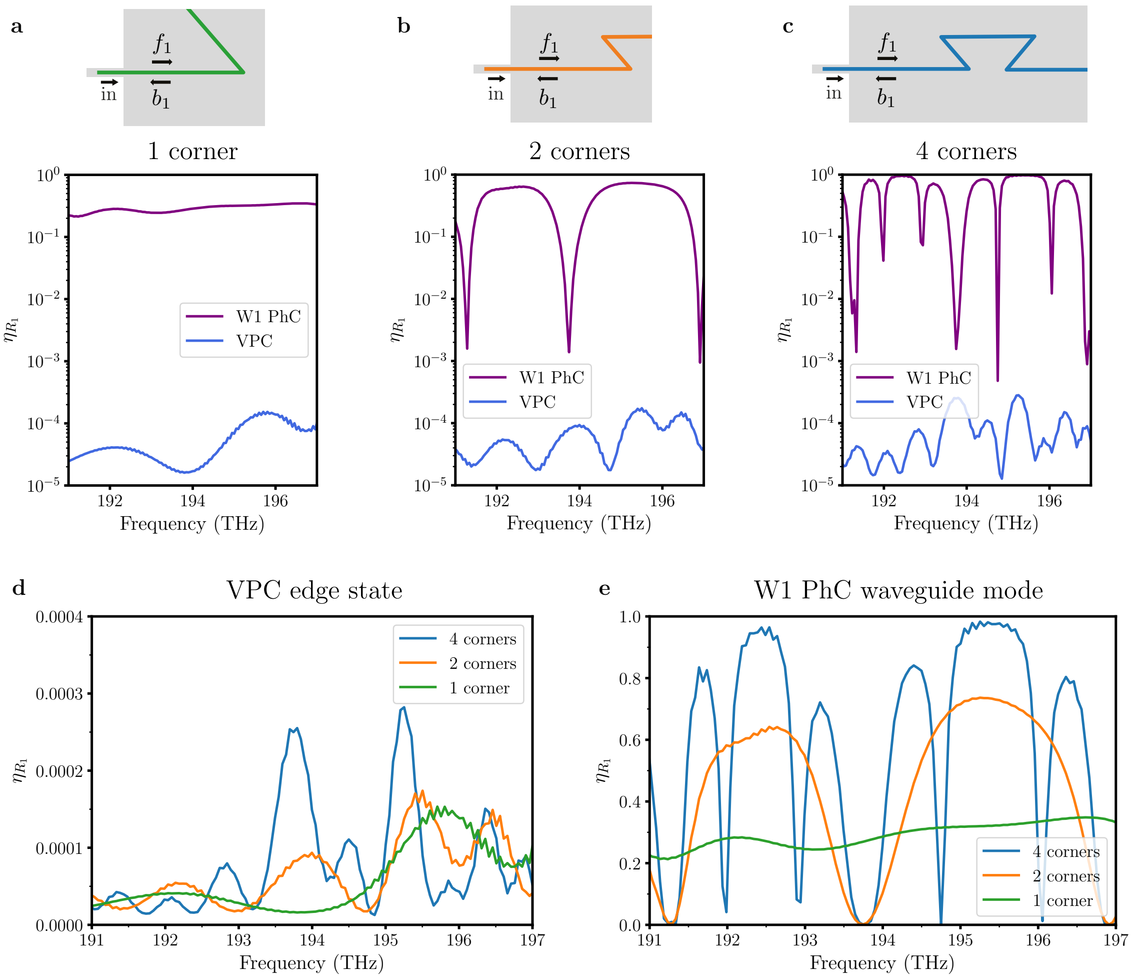

Figure S7. Numerically calculated energy ratio $\eta_{R_1} = \frac{B_1}{F_1}$ of the backward (B_1) and forward (F_1) propagating mode for different configurations of the guiding channel. A schematic of the simulation setup is shown above the corresponding ratio of a VPC edge state and W1 waveguide mode for **a**, a single 120° corner, **b**, two corners (Z-shaped defect) and **c**, four corners (Ω-shaped defect), where the mode amplitudes f_1 and b_1 are extracted from an area of 20 × 20 unit cells. To visualize the four orders of magnitude difference, the retrieved energy ratio is plotted in logarithmic scale. **d**, Comparison between the three different scenarios in **(a-c)** for the VPC edge state, and **e**, the W1 PhC waveguide mode.
